# Supplementary material for: The transmission mechanism of the housing price fluctuations on the global value chain position of manufacturing-evidence from China
Source: PLoS One. 2020 Feb 28;15(2):e0228598. doi: 10.1371/journal.pone.0228598 (PMC7048277; doi:10.1371/journal.pone.0228598)
Supplement: S1 File — (DOCX) [file pone.0228598.s001.docx]

1. The name of housing price is 𝑎𝑣𝑒𝑟𝑎𝑔𝑒 𝑠𝑎𝑙𝑒𝑠 𝑝𝑟𝑖𝑐𝑒 𝑜𝑓 𝑐𝑜𝑚𝑚𝑒𝑟𝑐𝑖𝑎𝑙 ho𝑢𝑠𝑒.
2. Globe value position is measured by GVC position index, among GVC equation, the name set of IV is Gross exports of intermediate products by origin of value added and final destination. the name set of FV is Foreign value added content of gross exports. The name set of E is Gross exports by origin of value added and final destination.
3. Human capital is calculated according to the equation: ${HC}_{it}=6E_{it}+9J_{it}+12S_{it}+12U_{it}+18.5$. $P_{it}$, $E_{it}$、$J_{it}$、$S_{it}$、$U_{it}$ and $P_{it}$ respectively represent of the population with education at the elementary, junior high, senior high, junior college and undergraduate, and postgraduate levels.
4. The name of resident consumption level is National consumer price index.
5. About resident consumption structure, China Statistical Yearbook divides residents' consumption into eight categories. We classify food, clothing, housing, transportation and telecommunication as survival consumption, classify household equipment and services, medical care, other goods and services as enjoyment consumption, and the culture, education and entertainment are classified as development consumption. And then, the ratio of these three types of consumption to the total consumption is the consumption structure.
6. R&D investment is measured by the ratio of R&D expenditures to the GDP. The data of R&D investment can be obtained from *Statistical Bulletin of China’s Science and Technology Funding*, these statistical bulletins can be found through searching at official website of Ministry of Science and Technology of the People’s Republic of China. GDP can be obtained from China Statistical Yearbook.
7. Economies of scale is calculated by the ratio of the total output value of manufacturing enterprises beyond the designated size to the number of enterprise units, among them, the total output value of manufacturing enterprises beyond the designated size and the number of enterprise units are from China industry economy statistical yearbook.
8. The data set of Foreign direct investment is Utilization of foreign capital.
9. Factor structure is calculated by the ratio of the total assets of the industrial enterprises beyond the designated size to the average number of employees, the data are also from China industry economy statistical yearbook.
10. Financing constraint is obtained by calculating the ratio of interest expenses to total liabilities of industrial enterprises beyond the designated size, the data are from China industry economy statistical yearbook.

Appendix 1：The research data

| Year | HP | RDI | PCL | HC | RCS |
| --- | --- | --- | --- | --- | --- |
| 2005 | 3168 | 1673.8 | 5771 | 7.83 | 39.61 |
| 2006 | 3367 | 2134.5 | 6416 | 8.04 | 40.23 |
| 2007 | 3864 | 2681.9 | 7572 | 8.19 | 39.79 |
| 2008 | 3800 | 3381.7 | 8707 | 8.27 | 38.15 |
| 2009 | 4681 | 4212.3 | 9514 | 8.38 | 39.53 |
| 2010 | 5032 | 5185.5 | 10919 | 8.35 | 40.31 |
| 2011 | 5357 | 6579.3 | 13134 | 8.85 | 40.18 |
| 2012 | 5791 | 7842.2 | 14699 | 8.94 | 40.32 |
| 2013 | 6237 | 9075.8 | 16190 | 9.05 | 41.36 |
| 2014 | 6324 | 10060.6 | 17778 | 9.04 | 39.44 |
| 2015 | 6793 | 10881.3 | 19397 | 9.14 | 40.19 |
| 2016 | 7476 | 12144 | 21228 | 9.15 | 40.97 |

| Year | GVC | Contr1 | Contr2 | Contr3 | Contr4 |
| --- | --- | --- | --- | --- | --- |
| 2005 | 0.1115 | 0.9256 | 638 | 35.4968 | 0.0146 |
| 2006 | 0.1181 | 1.0484 | 670 | 39.5756 | 0.0188 |
| 2007 | 0.1403 | 1.2031 | 783 | 44.829 | 0.0205 |
| 2008 | 0.1641 | 1.1909 | 952 | 48.8035 | 0.0228 |
| 2009 | 0.2098 | 1.2623 | 918 | 55.9033 | 0.0186 |
| 2010 | 0.1802 | 1.5426 | 1088 | 62.1164 | 0.0184 |
| 2011 | 0.1742 | 2.5929 | 1176 | 73.7182 | 0.0231 |
| 2012 | 0.1979 | 2.7032 | 1132 | 81.0624 | 0.0259 |
| 2013 | 0.206 | 2.9192 | 1187 | 88.9296 | 0.0244 |
| 2014 | 0.2191 | 2.9295 | 1197 | 95.8962 | 0.0248 |
| 2015 | 0.2525 | 2.8967 | 1262 | 104.6952 | 0.0225 |
| 2016 | 0.2709 | 3.0613 | 1260 | 114.5964 | 0.0206 |
