# Supplementary material for: The transmission mechanism of the housing price fluctuations on the global value chain position of manufacturing-evidence from China
Source: PLoS One. 2020 Feb 28;15(2):e0228598. doi: 10.1371/journal.pone.0228598 (PMC7048277; doi:10.1371/journal.pone.0228598)
Supplement: Attachment — Submitted filename: Response to Reviewers.docx [file pone.0228598.s002.docx]

Dear Editors and Reviewers:

Thank you for your letter and for the reviewers’ comments concerning our manuscript entitled “The Transmission Mechanism of the Housing Price Fluctuations on the Global Value Chain Position of Manufacturing--Evidence from China” (PONE-D-19-32876). Those comments are all valuable and very helpful for revising and improving our paper, as well as the important guiding significance to our researches.

We have revised the manuscript, according to the comments and suggestions of reviewers and editor, and responded, point by point to, the comments as listed below. Revised portion are marked in red in the paper.

Looking forward to hearing from you soon.

Kind regards,

Guangping Liu

Replies to Reviewers and Editor

First of all, we thank both reviewers and editor for their positive and constructive comments and suggestions.

**Reviewer #1:**

**Question:** Your paper analysis the effect of HP on GVC and get good conclusions and put forward excellent suggestions, which help us to understand the relationship between HP and GVC. But GVC may affect the HP. So, you may consider the endogeneity of the model if possible.

**Answer:** After careful thinking and analysis, we believe that GVC will not affect HP.

China is currently undergoing rapid urbanization. Land in China is monopolized by the government. The land, population are the main determinants of China's house price rise. the number of Chinese manufacturing enterprises participating in the division of global value chains is a very small number of enterprises. The changes in technology, economy, and talents of a few companies have negligible effects on housing prices.

**Reviewer #2:**

1. **Question:** Fig.1, table.2 should be marked with units.

**Answer:** We have marked units for Fig.1 and table.2 (now is table 3).

2. **Question:** The characters of "house price" in China should be explained. The story in China is quite special, housing price raised rapidly since housing reform in 1998, especially during 2005-2016. Statistics show that China's household real estate accounts for 69% of total assets. The author is suggested to explain these backgrounds for the readers to better understand the situation.

**Answer:** We have briefly explained some of the characteristics of "housing price" and added relevant information in the revised manuscript.

3. **Question:** The suggestions in the conclusion part can be further improved in terms of operability and innovation. For example, what other ways can we improve the level of human capital in addition to improving treatment? Can we consider career development and so on. Suggest the author to put forward more targeted and operational suggestions.

**Answer:** Thank you very much for the suggestion. We have improved the suggestions in the conclusion part to enhance both innovativeness and operability.

**Reviewer #3:**

1. **Question:** Abstract should be rewritten

**Answer:** Considering the Reviewer’s suggestion, we have rewritten the abstract to make it more informative and read more smoothly.

2. **Question:** Introduction part should be restructured.

Answer: We have revised Introduction part clearly illustrating the contribution of the study and supplementing the background of the research.

3. **Question:** Literature Review: The authors are highly recommended to take some time and prepare a well-structured table for the review of mainstream and other theoretic systems.

**Answer:** Thank you very much for the suggestion. We have added table for the review of mainstream and other theoretic systems in the revised manuscript (Table 2).

4. **Question:** Before you implement your empirical research, you should give some classical economics theories which support your conclusion. In fact, using real data, we can verify any correlations in the real word without any true theories base though these relationships are whimsical.

**Answer:** Thank you very much for the suggestion. Actually, in the research hypothesis 1 and 2 part, we put forward research hypotheses based on some theories such as labour mobility theory, the crowding out effect theory and wealth effect theory of housing prices on household consumption. In the research hypothesis 3 part, we added a paragraph to introduce how housing price fluctuations can affect R&D investment, among them, we mentioned some theories which are wealth effect, collateral effect and crowding out effect.
